# Supplementary material for: Regulatory Networks of Flowering Genes in Angelica sinensis during Vernalization
Source: Plants (Basel). 2022 May 19;11(10):1355. doi: 10.3390/plants11101355 (PMC9144295; doi:10.3390/plants11101355)
Supplement: Supplementary file 1 [file plants-11-01355-s001.zip › plants-1687618-supplementary.pdf]

Figure supplementary legends

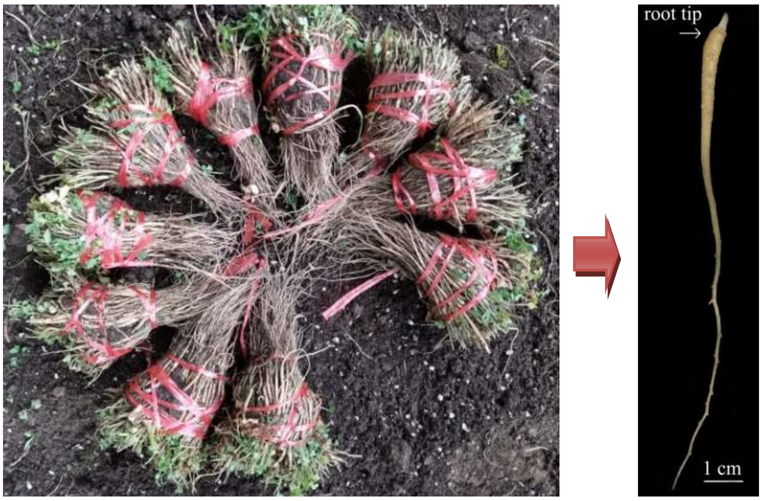

Figure S1. Morphological characteristic of *A. sinensis* seedlings.

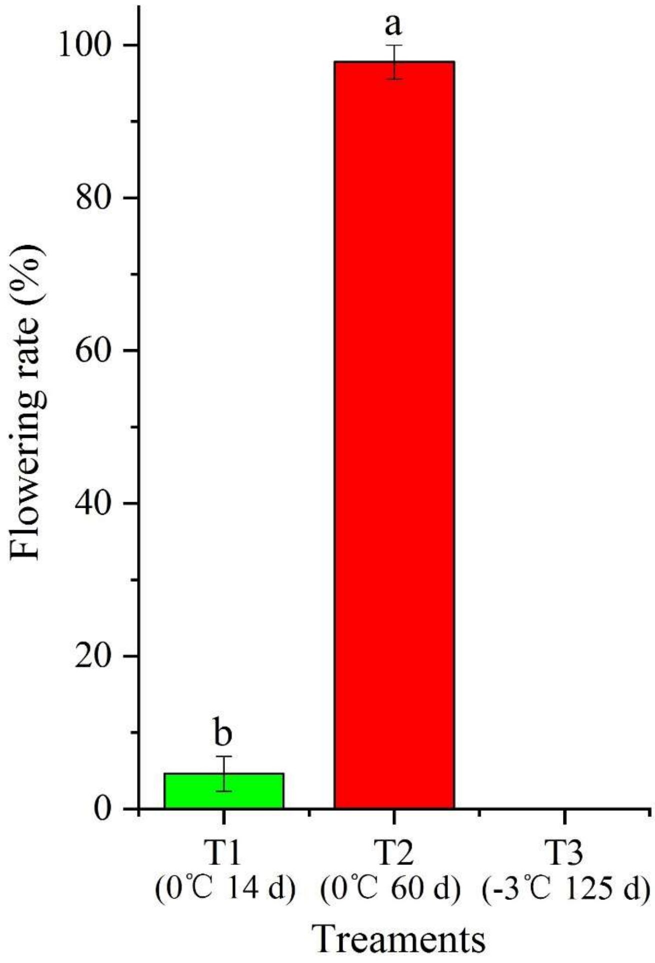

Figure S2. Flowering rate of *A. sinensis* after the seedlings stored at T1, T2 and T3. Different letters represent a significant difference ($p < 0.05$) at different treatments.

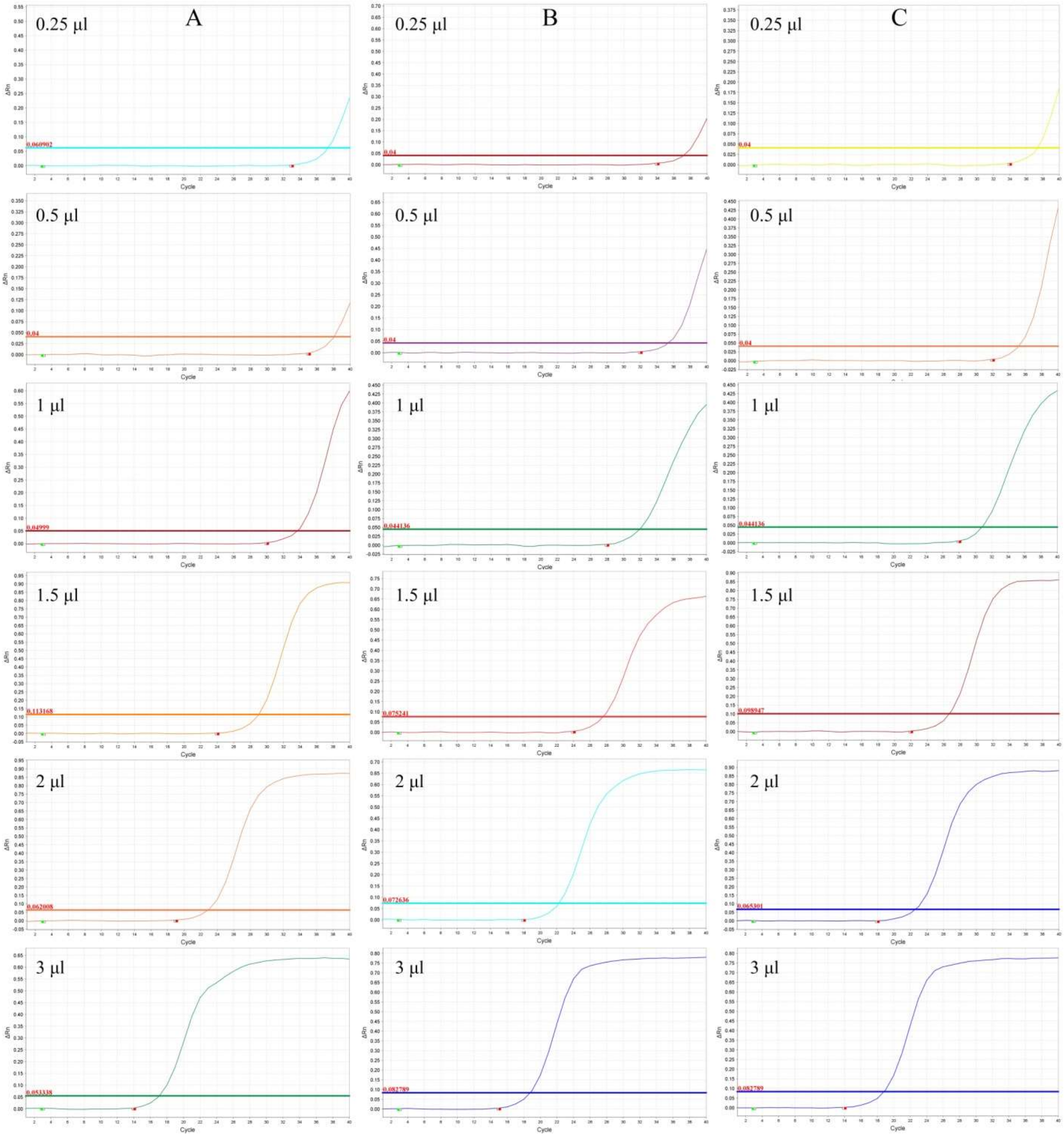

Figure S3. The cycle threshold (Ct) values of *ACT* gene at different volumes (0.25, 0.5, 1.0, 1.5, 2.0 and 3.0 µL) via PCR amplification with three replications.

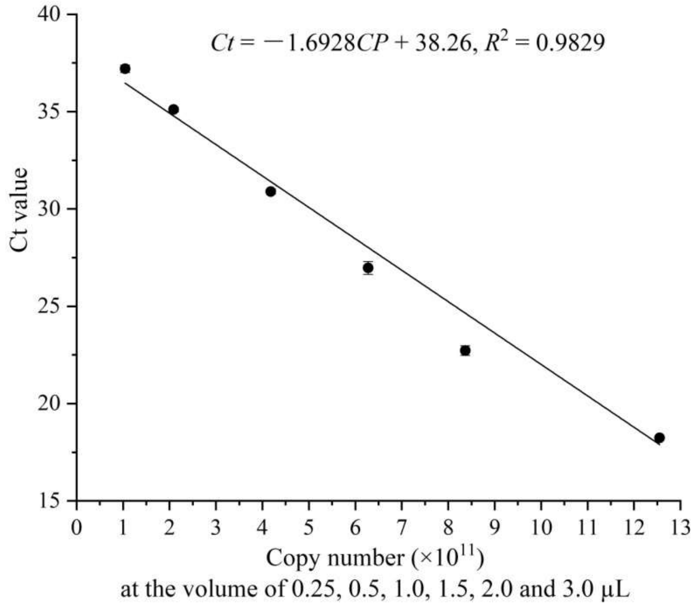

Figure S4. The standard curve of *ACT* gene.

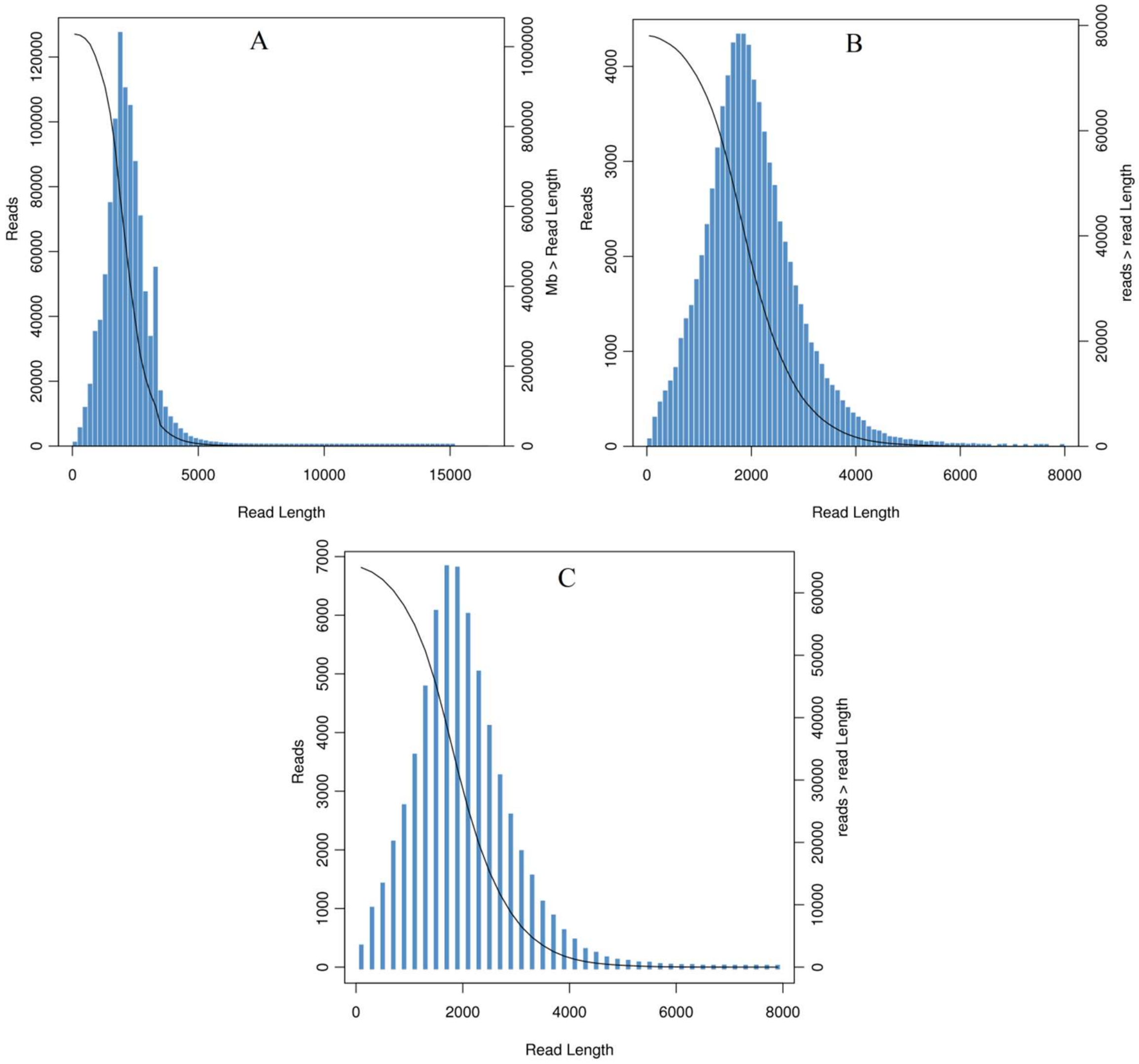

Figure S5. Number and length distribution of high-fidelity reads (A), polished high-quality isoforms (B) and full-length isoforms (C).

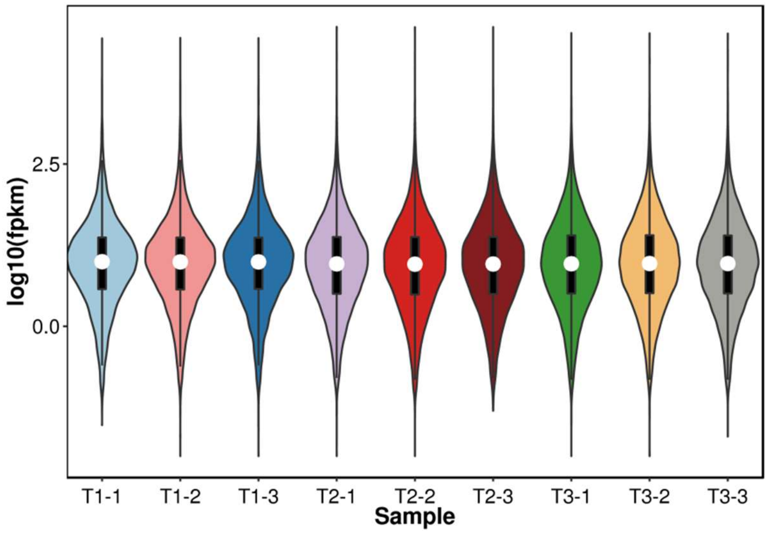

Figure S6. Violin plot of expression in T1, T2 and T3.

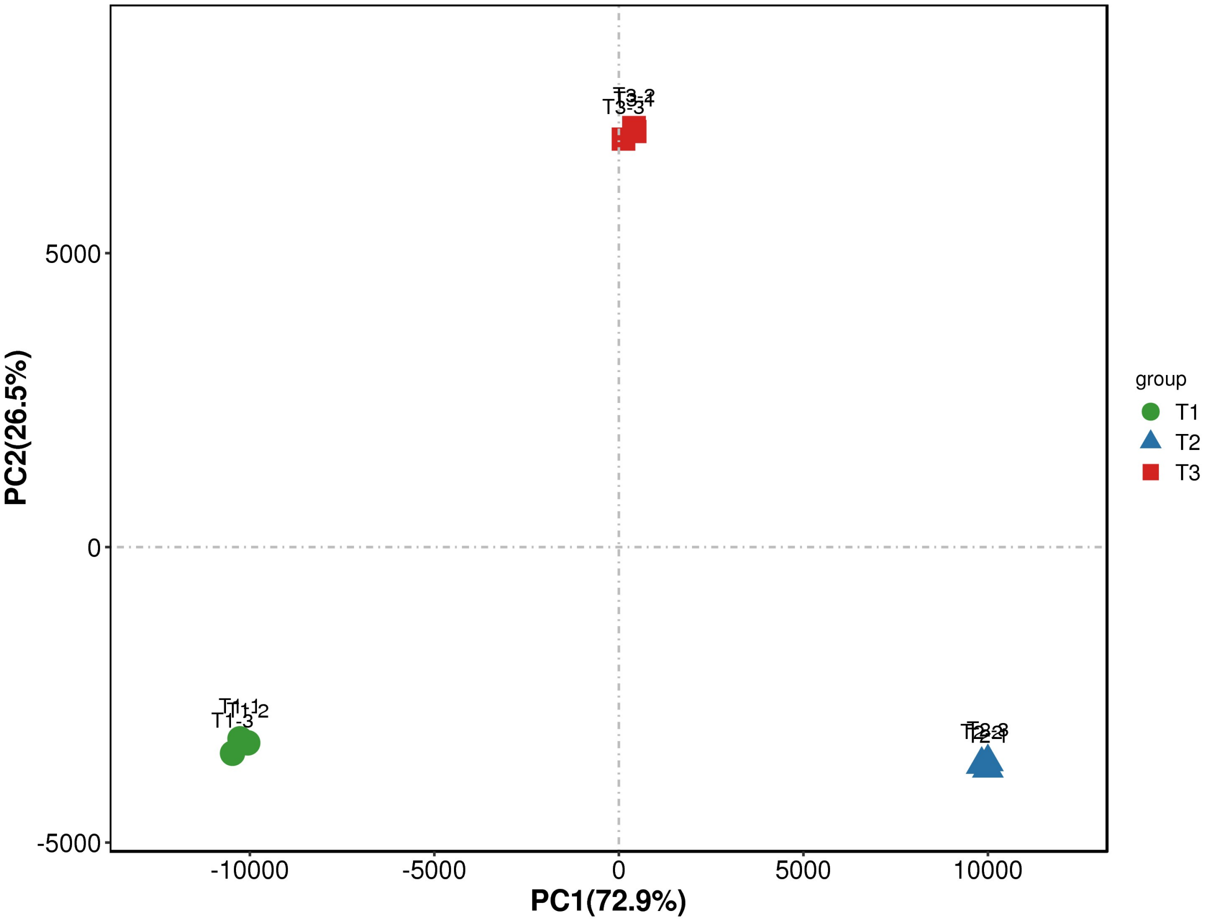

Figure S7. PCA analysis of T1, T2 and T3.

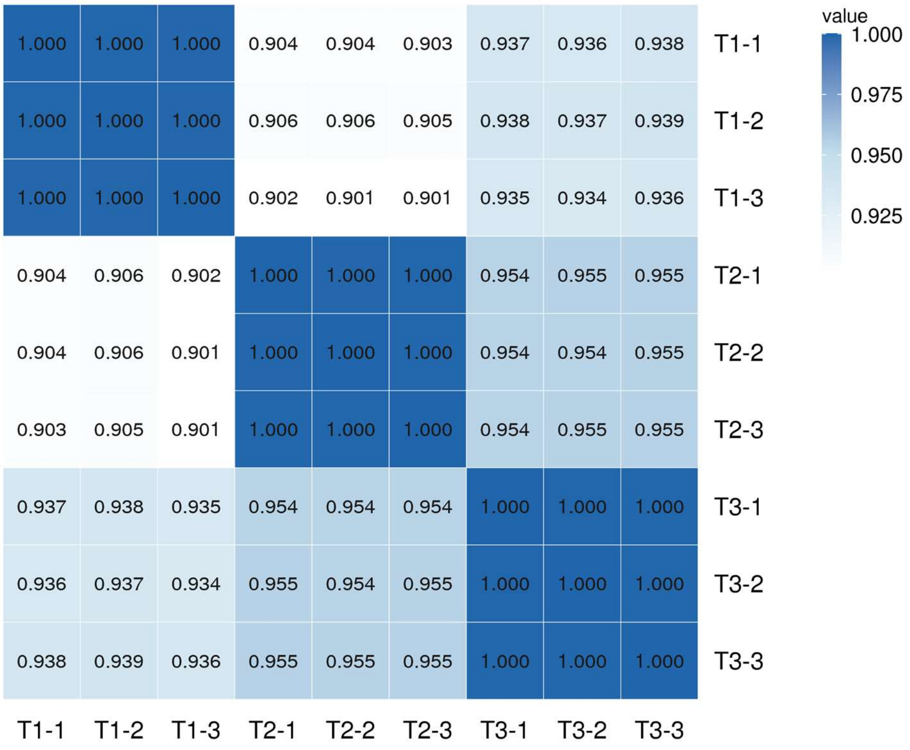

Figure S8. Pearson correlation analysis between T1, T2 and T3.

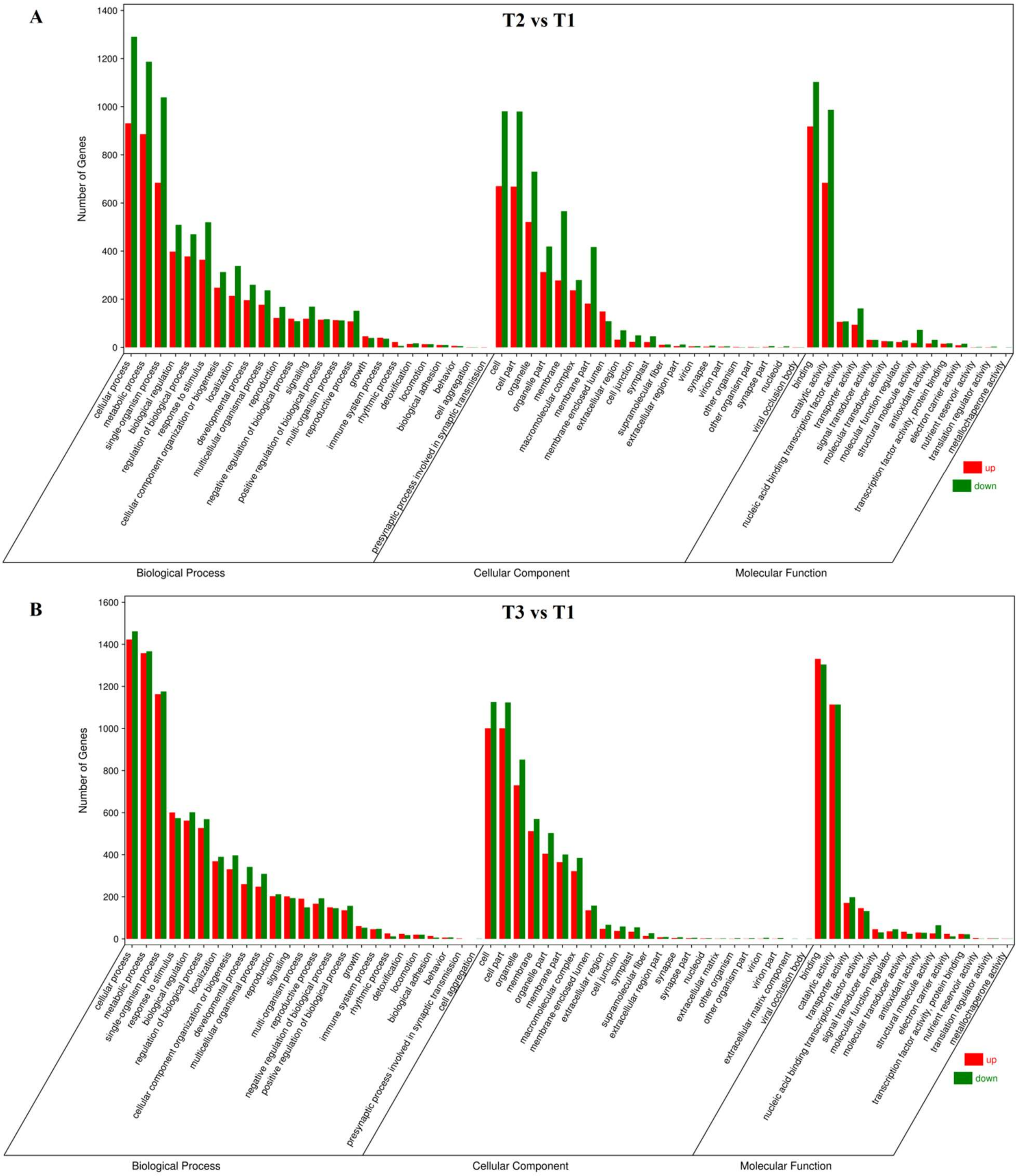

Figure S9. Gene Ontology (GO) enrichment of DEGs at T2 vs. T1 and T3 vs. T1.

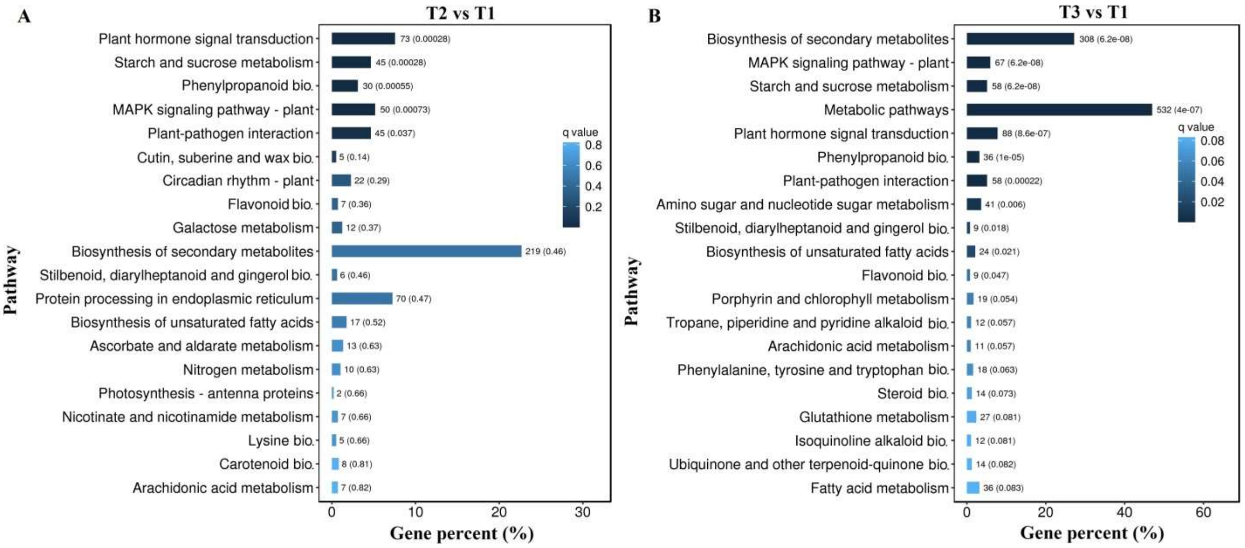

Figure S10. KEGG enrichment of DEGs at T2 vs. T1 and T3 vs. T1.

Table supplementary legends

Table S1. Sequences of primer used in qRT-PCR validation.

Gene name	Primer sequences (5' to 3')	Amplicon size (bp)
ACT	Forward: TGGTATTGTGCTGGATTCTGGT<br>Reverse: TGAGATCACCACCAGCAAGG	109
**FLC expression (15)**		
VIL1	Forward: TAAGGACGCTCGACGTGTTG<br>Reverse: TAGTTGAAACGCCACTCGCT	158
VIL2	Forward: TCAGATAGGTCCCCACGGAG<br>Reverse: CTGCCCCAATGGTGGAATCT	196
VRN1-L	Forward: GCGTATGTACTACCACCCACC<br>Reverse: TTGCTTAACCCGACCCGAC	106
FCA	Forward: CTTCAACACCGGGTGGTTCT<br>Reverse: TTGCGGCTTCGACTGGTTAT	122
FLK	Forward: GGGATAAACGCTGTTGGTGG<br>Reverse: TAGGAGGCATGTCTGCAGGT	106
FPA	Forward: TTGGTGAGCTTGAGAGCGTT<br>Reverse: GATTGCCCGCGACAGAAAAC	123
FY	Forward: TGCGATGCCGCTATCCATAC<br>Reverse: TGTTGGAGCTGCTGAGCATT	174
FLD	Forward: AGGCTACCACCAAGCGTTAC<br>Reverse: GGCAAGCACAAGAGTGTGTG	153
LD	Forward: ATAGTGGCTGGATGGTTGGC<br>Reverse: CAGCATCAGGCAACTGTTCG	198
LHP1	Forward: CTGTTCGTCGAAAGCGTGTT<br>Reverse: TACGCTTCCGCGATCTATGC	174
MAF1	Forward: GTGGTTCTTTGCTGGCTACC<br>Reverse: TAACAACGCGCTTAAGCTTTCT	171
VIP4	Forward: ACTACGAGTCCCGACGTTCT<br>Reverse: CGTCGGGATGATTTCGAAGC	151
ATX2	Forward: GATTTCGTGCTGAGCTTGGTG<br>Reverse: TCATCCTCTCCACTTGCACT	122
ARP6	Forward: TGGAGCCACAGGACAACATAC<br>Reverse: ATCTCCGGCACAAGGAAACG	138
FRL3	Forward: ATGCCAGCTCCTTGGTTACG<br>Reverse: GTCACGAAGACCGAAAGCAC	112
**Sucrose metabolism (3)**		
TPS1	Forward: ACGCAGGGAGAATGGATGAC<br>Reverse: AACGGGGTGTCCTTTCAGTG	103
SUS3	Forward: AGGGAACTGGCAAACCTTGT<br>Reverse: ACCGATAGAGCTCGCCATTG	181
SPS	Forward: GACGGTGAGAGGAATGATGGT<br>Reverse: TGTGTCCCCTTCATGCCAAC	119
**Hormone response (5)**		
GID1B	Forward: AGCCCCAGACAACGAAACTC	118

	Reverse: GGAAGACGAATGGGCAAAGC	
RAP2-7	Forward: ACGCCGGTTGATTATCGGTT<br>Reverse: CCACGATACTGCGAGCTTCT	116
IAA13	Forward: CTGCGATTGGACGAAAGGTG<br>Reverse: CCCCTCCATGCTTGATCGTG	122
ARF1	Forward: ACTGTTTGCCCCCATTGGAT<br>Reverse: AGCATCACCAGCAACCAACT	179
AHK3	Forward: CGAACACATGGGGGTACAGG<br>Reverse: CCGACTGCTGGTTGATTTGC	184
**Circadian clock (2)**		
ELF3	Forward: TGATGCCGAGTCTCGTGAAG<br>Reverse: CTGTGCCGGACATCGAATCT	107
COR27	Forward: GGATTTGCATGGATCGCTGTC<br>Reverse: GTCCAGCAACCATCACGAAG	123
**Downstream floral integrators and meristem identity (6)**		
FTIP3	Forward: GGACAAGGTTCCCGGATGTT<br>Reverse: TCCTTGTTGGGTGCAACTCT	181
FTIP7	Forward: TGGGAAGGTGGTGTTCGATG<br>Reverse: ATCACTATGCCATGCCTCGG	163
SOC1	Forward: GAGAAGGCTTGGGATCGTGTT<br>Reverse: CTGCATTATCTGCTGCGAGG	159
AGL65	Forward: AGGTCTCACCAGATTTTGCCG<br>Reverse: ATACGGGTTGGGACTCTTGC	100
SPL1	Forward: AGCATCGCACCAAGACTCTC<br>Reverse: TCCTATCTGTGCGGCTCTCT	113
SOK2	Forward: ACACTGCTCCAAACACTCCAT<br>Reverse: GTTGTCGTTTGCCTTGTCGG	176
**Cold response (9)**		
PYL3	Forward: GCTGCTCATCAGTGCTCTTCT<br>Reverse: TTTTCGTGGCTGGAAGACCT	193
ERF110	Forward: ACCACTACAGCCACCTTGTC<br>Reverse: ACTCTAGCAGCTTTGTGCGG	137
ETR2	Forward: TGCAGTGCTAGAAGAGTCGC<br>Reverse: GAATTCCTTGCGTGGGTAGC	108
COR413PM1	Forward: AGGCGCGATGATCCTTATCTG<br>Reverse: GAGGCGCGTATGTGTTCCTG	129
CRPK1	Forward: GAAGCTGAGATGTTGCGGTTC<br>Reverse: TTGCGAGGTGTAACGCCTAT	165
MPK3	Forward: TGGCGCTTACGGAATTGTCT<br>Reverse: AGTTCGCTTCGCATCCATGT	109
SRK2E	Forward: GTATGCATCCGGAGGAGAGC<br>Reverse: TCGCGGTGACATACTTGCAT	135
PP2CA	Forward: CGTTCTCCGGTGGAAGTCAT<br>Reverse: CCTTGCACTTCATCGCAACC	186
HAB1	Forward: ATATTAGCCAGCGACGGTCTG<br>Reverse: GTAACCGGCCCGTTCTTTCT	104

Table S2. The sequence details of the isoforms involved in the 104 co-expressed genes at T2 vs. T1 and T3 vs. T1. Note, the Table S2 is shown as a separate document of .txt format.

Table S3. DEGs involved in FLC expression at T2 vs. T1 and T3 vs. T1.

**Gene name**	**Swiss-Prot ID**	**Protein name**	**FPKM value**	
			**T2/T1**	**T3/T1**
**Inhibiting FLC expression**				
VIL1	Q9LHF5	VIN3-like protein 1	-1.28	-0.40
VIL2	Q9SUM4	VIN3-like protein 2	-1.43	-1.18
VRN1-L	A0A6P4B193	B3 domain-containing transcription factor VRN1-like	-0.55	2.07
FCA	O04425	Flowering time control protein FCA	0.85	2.47
FLK	Q9SR13	Flowering locus K homology domain	0.21	-0.42
FPA	Q8LPQ9	Flowering time control protein FPA	1.04	-1.15
FY	Q6NLV4	Flowering time control protein FY	0.29	-0.19
FLD	Q9CAE3	Protein FLOWERING LOCUS D	-0.77	-0.35
LD	Q38796	Homeobox protein LUMINIDEPENDENS	-1.17	-1.71
LHP1	Q944N1	Chromo domain protein LHP1	0.98	0.80
MAF1	Q7ZWL6	Repressor of RNA polymerase III transcription MAF1 homolog	-1.41	-1.33
**Activating FLC expression**				
VIP4	Q9FNQ0	Protein LEO1 homolog	1.36	0.31
ATX2	P0CB22	Histone-lysine N-methyltransferase ATX2	0.31	-8.75
ATX4	Q9SUE7	Histone-lysine N-methyltransferase ATX4	0.11	-1.55
ATX5	Q8GZ42	Histone-lysine N-methyltransferase ATX5	0.34	-0.86
ARP6	Q8LGE3	Actin-related protein 6	-1.06	-1.10
PIE1	Q7X9V2	Protein PHOTOPERIOD-INDEPENDENT EARLY FLOWERING 1	0.37	1.78
FRL3	Q67ZB3	FRIGIDA-like protein 3	1.27	-1.48
FES1	Q84VG7	Protein FRIGIDA-ESSENTIAL 1	1.11	1.47
SUF4	Q9C5G0	Protein SUPPRESSOR OF FRI 4	2.31	1.17
**Interacting FLC expression**				
CLF	P93831	Histone-lysine N-methyltransferase CLF	-0.02	-0.56
FIE2	Q6ZJX0	Polycomb group protein FIE1	1.03	-0.92

Table S4. DEGs involved in sucrose metabolism at T2 vs. T1 and T3 vs. T1.

**Gene name**	**Swiss-Prot ID**	**Protein name**	**FPKM value**	
			**T2/T1**	**T3/T1**
TPS1	Q9SYM4	Alpha, alpha-trehalose-phosphate synthase 1	1.35	1.04
TPS5	O23617	Alpha, alpha-trehalose-phosphate synthase 5	-1.50	-1.42
TPS7	Q9LMI0	Probable alpha, alpha-trehalose-phosphate synthase 7	0.59	1.61
TPS10	O80738	Probable alpha, alpha-trehalose-phosphate synthase 10	3.22	3.12

SUS2	O24301	Sucrose synthase 2	-1.12	-1.55
SUS3	Q9M111	Sucrose synthase 3	1.11	-1.70
SPS	Q43845	Probable sucrose-phosphate synthase	-1.55	-2.92
SPS1	O22060	Probable sucrose-phosphate synthase 1	-1.00	-2.45
PGMP	Q9SCY0	Phosphoglucomutase, chloroplastic	-1.59	-1.00
PGM1	Q9ZSQ4	Phosphoglucomutase, cytoplasmic	-1.83	-3.19
BAM1	Q9LIR6	Beta-amylase 1, chloroplastic	-2.86	-1.88
BAM3	O23553	Beta-amylase 3, chloroplastic	-2.36	-1.67

Table S5. DEGs involved in hormone response at T2 vs. T1 and T3 vs. T1.

Gene name	Swiss-Prot ID	Protein name	FRPM value	
			**T2/T1**	**T3/T1**
**Gibberellin**				
GID1B	Q9LYC1	Gibberellin receptor GID1B	-0.28	5.56
**Ethylene**				
RAP2-3	P42736	Ethylene-responsive transcription factor RAP2-3	-2.44	-2.39
RAP2-7	Q9SK03	Ethylene-responsive transcription factor RAP2-7	-2.07	-0.74
**Auxin**				
GH3.6	Q9LSQ4	Indole-3-acetic acid-amido synthetase GH3.6	-2.67	-3.50
LAX2	Q9S836	Auxin transporter-like protein 2	-3.68	-5.37
IAA8	Q38826	Auxin-responsive protein IAA8	-1.46	-1.13
IAA9	Q38827	Auxin-responsive protein IAA9	-1.46	-1.25
IAA12	Q38830	Auxin-responsive protein IAA12	-2.60	-2.52
IAA13	Q38831	Auxin-responsive protein IAA13	-1.04	0.06
IAA27	Q9ZSY8	Auxin-responsive protein IAA27	-3.31	-4.02
ARF1	Q8L7G0	Auxin response factor 1	-1.26	-1.07
ARF4	Q9ZTX9	Auxin response factor 4	-1.33	-1.58
ARF5	P93024	Auxin response factor 5	-2.21	-1.54
ARF6	Q9ZTX8	Auxin response factor 6	1.02	1.04
ARF19	Q0D9R7	Auxin response factor 19	-1.75	-1.93
**Cytokinin**				
AHK3	Q9C5U1	Histidine kinase 3	2.42	2.25
AHP1	Q9ZNV9	Histidine-containing phosphotransfer protein 1	1.58	-0.12
ARR2	Q9ZWJ9	Two-component response regulator ARR2	-1.61	0.71

Table S6. DEGs involved in circadian clock at T2 vs. T1 and T3 vs. T1.

Gene name	Swiss-Prot ID	Protein name	FPKM value	
			**T2/T1**	**T3/T1**
ELF3	O82804	Protein EARLY FLOWERING 3	1.58	1.58
COR27	Q8L8T7	Cold-regulated protein 27	1.68	1.80

Table S7. DEGs involved in downstream floral integrators and meristem identity at T2 vs. T1 and T3 vs. T1.

Gene name	Swiss-Prot ID	Protein name	FRPM value	
			T2/T1	T3/T1
FTIP3	Q9M2R0	FT-interacting protein 3	2.40	1.55
FTIP7	Q60EW9	FT-interacting protein 7	1.27	1.59
SOC1	O64645	MADS-box protein SOC1	1.00	0.32
AGL65	Q7X9I0	Agamous-like MADS-box protein AGL65	-1.74	1.25
SPL1	Q9SMX9	Squamosa promoter-binding-like protein 1	1.19	-1.18
SPL6	Q94JW8	Squamosa promoter-binding-like protein 6	1.90	-2.31
AP2	P47927	Floral homeotic protein APETALA 2	-0.03	1.65
AP2-1	B8AXC3	APETALA2-like protein 1	-2.43	-1.53
AP2-2	B8AMA9	APETALA2-like protein 2	-1.51	-1.14
AP2-3	B8B8J2	APETALA2-like protein 3	-1.29	-0.24
AIL5	Q6PQQ3	AP2-like ethylene-responsive transcription factor AIL5	-1.84	1.22
ANT	Q38914	AP2-like ethylene-responsive transcription factor ANT	-1.36	-1.92
At2g41710	Q8GWK2	AP2-like ethylene-responsive transcription factor At2g41710	-1.84	-1.57
TEM1	Q9C6M5	AP2/ERF and B3 domain-containing transcription repressor TEM1	1.55	1.28
SOK2	Q9LX14	Protein SOSEKI 2	-3.63	3.03

Table S8. DEGs genes involved in cold response at T2 vs. T1 and T3 vs. T1.

Gene name	Swiss-Prot ID	Protein name	FRPM value	
			T2/T1	T3/T1
PYL3	Q6EN42	Abscisic acid receptor PYL3	1.13	1.30
PYL4	O80920	Abscisic acid receptor PYL4	1.15	7.36
ERF3	Q9SXS8	Ethylene-responsive transcription factor 3	1.30	1.16
ERF5	Q9LW48	Ethylene-responsive transcription factor 5	2.52	4.58
ERF010	Q9FH94	Ethylene-responsive transcription factor ERF010	1.84	2.25
ERF011	Q9SNE1	Ethylene-responsive transcription factor ERF011	1.28	1.42
ERF13	Q8L9K1	Ethylene-responsive transcription factor 13	1.93	0.65
ERF110	Q70II3	Ethylene-responsive transcription factor ERF110	1.79	3.32
ERF118	Q9CA27	Ethylene-responsive transcription factor ERF118	2.00	1.23
ETR2	Q0WPQ2	Ethylene receptor 2	2.17	1.92
COR413PM1	Q9XIM7	Cold-regulated 413 plasma membrane protein 1	1.10	-2.42
COR413PM2	Q9SVL6	Cold-regulated 413 plasma membrane protein 2	4.07	-3.29
CRPK1	Q93YN1	Cold-responsive protein kinase 1	-3.00	1.17
MPK3	Q39023	Mitogen-activated protein kinase 3	1.61	3.47
MPK9	Q9LV37	Mitogen-activated protein kinase 9	1.38	-1.07
MPK16	Q8W4J2	Mitogen-activated protein kinase 16	1.30	2.22
YDA	Q9CAD5	Mitogen-activated protein kinase kinase kinase YODA	-1.41	-2.57
SRK2E	Q940H6	Protein phosphatase 2C 70	1.25	0.48

PP2CA	P49598	Protein phosphatase 2C 3	-2.87	-4.23
HAB1	Q9CAJ0	Protein phosphatase 2C 16	-1.90	-5.44
HAB2	Q9LNP9	Protein phosphatase 2C 7	-3.51	-3.64
KAPP	P46014	Protein phosphatase 2C 37	1.48	-1.35
PPC6-1	Q9M8R7	Probable protein phosphatase 2C 33	1.01	1.31
PPC6-7	Q0WRB2	Probable protein phosphatase 2C 73	-1.19	-1.24
PP2C27	P93006	Probable protein phosphatase 2C 27	-1.88	1.48
PP2C38	Q9LHJ9	Probable protein phosphatase 2C 38	-2.25	1.39
At2g30020	O80871	Probable protein phosphatase 2C 25	1.75	2.70
Os02g0799000	Q69QZ0	Probable protein phosphatase 2C 27	2.87	2.02
At3g15260	Q9LDA7	Probable protein phosphatase 2C 39	-3.08	-3.72
At3g16560	Q9LUS8	Probable protein phosphatase 2C 40	1.61	2.82
At3g62260	Q3EAF9	Probable protein phosphatase 2C 49	-1.46	0.34
Os06g0651600	Q67UP9	Probable protein phosphatase 2C 58	1.24	-0.19
At4g31860	Q9SZ53	Probable protein phosphatase 2C 60	0.06	-0.99
TGA2.2	Q6IVC3	Transcription factor TGA2.2	-2.00	-0.96
CDC5	P92948	Cell division cycle 5-like protein	3.83	9.48
